# Evaluation of plasma carnitine status in patients diagnosed with juvenile idiopathic arthritis

**DOI:** 10.55730/1300-0144.5366

**Published:** 2022-01-25

**Authors:** Ayşe Ciğdem AKTUĞLU ZEYBEK, Ertuğrul KIYKIM, Kenan BARUT, Tanyel ZÜBARİOĞLU, Mehmet Şerif CANSEVER, Özgür KASAPÇOPUR

**Affiliations:** 1 Department of Pediatrics Division of Nutrition and Metabolism, Cerrahpaşa Medical Faculty, İstanbul University-Cerrahpaşa, İstanbul, Turkey; 2 Department of Pediatrics Division of Rheumatology, Cerrahpaşa Medical Faculty, İstanbul University-Cerrahpaşa, İstanbul, Turkey; 3 Department of Medical Documentation and Techniques Division of Medical Laboratory Technique, The Vocational School of Health Services, İstanbul University-Cerrahpaşa, İstanbul, Turkey

**Keywords:** Autoinflammation, carnitine deficiency, juvenile idiopathic arthritis, fatty acid metabolism

## Abstract

**Background/aim:**

Juvenile idiopathic arthritis (JIA) is the most common rheumatic disease in childhood and manifests mainly as autoinflammation of the joints and other tissues. Several treatment options such as nonsteroidal antiinflammatory drugs, methotrexate, and intra-articular steroids are widely used to relieve and improve this inflammation. Secondary carnitine deficiency can be detected in chronic diseases by either renal loss or increased demand. While carnitine status can be associated with several conditions, in the present study our aim is to determine the levels of free carnitine and acyl-carnitine in Turkish JIA patients.

**Materials and methods:**

One hundred and fourteen patients diagnosed with juvenile idiopathic arthritis and 50 healthy individuals who served as the control group were included in the study. A fasting blood sample was collected from the children in both groups to determine free carnitine and acylcarnitine ester by quadripole electrospray tandem mass spectrometry (ESI-MS/MS).

**Results:**

Screening of acyl-carnitine profile revealed free carnitine, C14, C14:2, C16, C16-OH, and C18 carnitine levels were higher (p < 0.0001, p < 0.0001, p < 0.001, p < 0.001, and p = 0.011, respectively), while C2, C3, C4, C6, C8, C10, C10:1, C10:2, C3DC, C4DC, C5DC, C4-OH, and C18:1-OH carnitine levels were lower (p < 0.0001) in JIA patients in comparison to the control group. Total acyl-carnitine levels (p < 0.001) and acyl-carnitine to free carnitine ratio (p < 0.001) were also lower in JIA patients than the control group. Free carnitine levels were significantly higher (48.05 ± 13.36 μmol/L) in patients under antiinflammatory drug therapy than those who did not receive any treatment (43.18 ± 7.96 μmol/L) (p = 0.004).

**Conclusion:**

In the present study we were not able to define secondary carnitine deficiency in JIA patients, although free carnitine and acyl-carnitine variations were detected in JIA patients. In conclusion, routine carnitine supplementation is not recommended in all patients with JIA.

## 1. Introduction

Juvenile idiopathic arthritis (JIA) consists of all arthritis without a known etiology, starting before 16 years of age and lasting at least 6 weeks long. Among rheumatic diseases of the childhood period, JIA is the most frequent disorder with a reported prevalence of 16 and 150 per 100,000 children [1].

The underlying mechanisms responsible for pathophysiology of JIA still remain unclear and poorly understood but seem to have both genetic and environmental components [2]. The clinical spectrum of the disease is mostly heterogeneous and shows a wide clinical diversity. Therefore, the JIA classification was based on clinical findings, family history, and laboratory examination including rheumatoid factor (RF), antinuclear antibodies (ANA), and HLA-B27. Juvenile idiopathic arthritis is classified within seven subgroups based on specific inclusion and exclusion criteria according to ILAR (The International League of Associations for Rheumatology) classification [3]. Treatment consists of pharmacotherapy, physical therapy, and physiological support. Various measures are used in activity evaluation in JIA, the most widely used scale is JADAS (Juvenile Arthritis Disease Activity Score). Activity criteria guide the treatment selection. The main goal of the treatment is to achieve complete control of inflammation and control the JIA activation [4,5].

L- Carnitine (β-hydroxy-γ-trimethylaminobutyric acid) is widely supplied in diet, mainly in foods from animal sources like red meat and milk. Carnitine can also endogenously be synthesized from two essential amino acids lysine and methionine [6]. In cases where carbohydrate storage is depleted, homeostasis of cellular energy metabolism essentially becomes dependent on mitochondrial fatty acid β-oxidation [6,7]. Carnitine plays an essential role in long-chain fatty acids’ (LCFAs) mitochondrial β-oxidation, particularly in steps of esterification and transport into mitochondria. In addition to its pivotal role in energy metabolism, carnitine is cytoprotective, neuroprotective, and neuromodulator. The protective effect of carnitine against oxidative stress has also been reported [8–10]. There is not sufficient data in the medical literature to claim that chronic low-to-moderate inflammation causes carnitine deficiency. However, in chronic diseases accompanied by systemic inflammation carnitine replacement resulted in amelioration in inflammation biomarkers. Chronic inflammation increases the release of inflammatory cytokines such as interleukin-6 and tumor necrosis factor-a which are responsible for the synthesis of systemic inflammation markers such as C-reactive protein (CRP) and serum amyloid A (SAA). In patients with chronic kidney disease and cardiovascular diseases, which is one of the disorders accompanied by inflammation, carnitine supplementation has been found to be effective in reducing serum CRP and SAA, two systemic inflammation markers. In conclusion, it could be suggested that there is a clear relationship between prolonged inflammation and carnitine deficiency [11–13].

Carnitine deficiencies can be classified as primary and secondary carnitine deficiency. Primary carnitine deficiency (PCD) is an inherited metabolic disorder of disturbed fatty acid oxidation [14]. Secondary carnitine deficiency (SCD) can be relevant to some inherited metabolic disorders, pharmacotherapy like valproate, zidovudine, and pivampicillin [11–18]. Secondary carnitine deficiency can also be associated with chronic diseases such as kidney disease, cirrhosis, diabetes mellitus, heart failure, and malabsorption [19–21]. Trauma, septicemia, and acute organ failure result in SCD by increasing carnitine demand [22–24].

Juvenile idiopathic arthritis is a clinical entity that mainly presents with autoinflammation in joints and other tissues. Nonsteroidal antiinflammatory drugs, methotrexate, intra-articular steroids are listed as treatment options used to improve the inflammatory process [25]. We hypothesized that SCD may be seen due to the increased demand in JIA patients. In this study, we aimed to determine plasma carnitine status in Turkish JIA patients.

## 2. Material and method

### 2.1. Study design and population

Our study is a cross-sectional study that was conducted with 114 JIA patients followed by the outpatient Pediatric Rheumatology clinic of Cerrahpaşa Medical Faculty Children’s Hospital. The diagnosis of JIA was made by a pediatric rheumatologist using ILAR criteria. Exclusion criteria were the absence of a definite diagnosis of JIA, treatment with carnitine, and refusal to participate in the study. Demographic and clinical data including age, sex, age at diagnosis, family history (including consanguinity, additionally affected siblings), classification of JIA, clinical findings (fatigue, muscle pain-cramps etc.), therapeutic agents, and response to medical treatment were recorded. A detailed physical examination was performed on all JIA patients. The JADAS measurement scale was used to evaluate the activity of JIA patients [5]. An age-matched group of 50 healthy individuals was included as a control group. In order to exclude any cause that may lead to inflammation, control group participants were selected from the population without any chronic disease, drug or substance use, and nutritional problems (obesity or malnutrition). Participants with a recent history of an acute illness or vaccination were also excluded from the control group.

In both groups, a fasting blood sample was collected for free carnitine (FC) and acylcarnitine (AC) esters by quadripole electrospray tandem mass spectrometry (ESI-MS/MS). Samples consisted of capillary blood collected on Whatman S&S 903 filter paper. The samples were dried at room temperature. Relevant AC butyl esters were extracted and derivatized from 1/3 inch dried blood samples and analyzed according to a protocol previously described by Schulze et al [22]. AC butyl esters were detected based on mass/ion ratios using ESI-MS/MS. An Agilent 1200 Series Autosampler and Water Micromass Quattro LC Likrom™ tandem mass spectrometry were used for the analyzes. The values of AC were checked using the screening function for positive ions mod m/z 85 parents. Quantitative values of signal intensities were calculated using Masslynx and Neolynx software (Ver 4.1). Both signal intensities and calculated concentrations were exported to spreadsheet software where abnormal concentration tagging and further analysis were performed [26,27].

The study protocol was designed according to the principles of Ethical Guidelines for Biomedical Research Involving Human Subjects as defined in the Declaration of Helsinki and was approved by the Ethical Committee of İstanbul University-Cerrahpaşa, Cerrahpaşa Medical Faculty (15.12.2020/83045809-604.01.02). All parents of the patients included in the present study gave informed consent.

### 2.2. Statistical analysis

Statistical analyses were performed using Statistical Package for Social Sciences version 26.0 (SPSS Inc., Chicago, IL, USA). The mean and standard deviation were used as descriptive statistics. Categorical variables were expressed as numbers and percentages. A one-sample t-test was carried out for the quantitative estimation of acyl-carnitine analyses. Comparison between the groups was carried out by independent sample t-test.

## 3. Results

One hundred fourteen patients with JIA were enrolled in the study. Data concerning clinical and demographic findings of the patients are shown in Table 1. Free carnitine and acyl-carnitine analyses in spot dried blood samples with ESI-MS/MS were performed in all patients. Evaluation of the acyl-carnitine profile revealed statistically increased levels of free carnitine, C14, C14:2, C16, C16-OH, and C18 carnitine (p < 0.0001, p < 0.0001, p < 0.001, p < 0.001, and p = 0.011 respectively), while C2, C3, C4, C6, C8, C10, C10:1, C10:2, C3DC, C4DC, C5DC, C4-OH, and C18:1-OH carnitine levels were lower (p < 0.001) in JIA patients in comparison the control group. Total acyl-carnitine levels (p < 0.001) and acyl-carnitine to free carnitine ratio (p < 0.001) were also lower in JIA patients than in the control group. Table 2 shows the acyl-carnitine profile of both groups.

In terms of pharmacotherapy, 95 patients were under antiinflammatory treatment and 19 were not receiving any treatment agent. Fourteen of 95 patients were using a combination of three antiinflammatory drugs (either etanercept, adalimumab, or anakinra was combined with methotrexate and steroid), 41 were using a combination of two antiinflammatory drugs (combination of etanercept, methotrexate, and steroid) and 40 were using one type of medication. No statistical difference in plasma FC and AC levels was detected between groups according to the treatment modality. On the other hand, FC levels were significantly higher in patients using antiinflammatory treatment than those without any treatment (48.05 ± 13.36 μmol/L, 43.18 ± 7.96 μmol/L, respectively) (p = 0.004).

## 4. Discussion

In this study, we evaluated the plasma carnitine status in patients diagnosed with JIA by using tandem mass spectrometry. Several studies reported the possible impact of inflammation resulting in carnitine deficiency [23,24]. However, it is the first study mentioning the carnitine status in JIA in the medical literature.

Carnitine deficiency and/or a decrease in plasma-free carnitine levels were not detected in children with JIA compared to the healthy control group in our study. On the contrary, free carnitine, myristoylcarnitine (C14), tetradecenoylcarnitine (C14:1), palmitoylcarnitine (C16), 3-hydroxypalmitoylcarnitine (C16-OH), octadecanoylcarnitine (C18) levels were prone to be elevated in children with JIA. In JIA patients C2 (Acetyl-), C3 (Propionyl-), C4 (Butyryl-), C6 (Hexanoyl-), C8 (Octanoyl-), C10 (Decanoyl-), C10:1 (Decenoyl-), C10:2 (Decadienoyl-), C3DC (Malonyl-), C4DC (Methylmalonyl-), C5DC (Glutaryl-), C4-OH (3-Hydroxy butyryl), and C18:1-OH (3-Hydroxy octadecenoyl-) carnitine levels were lower in comparison to the control subjects. On the other hand, total acylcarnitine levels and acyl to free carnitine ratio were also found to be lower in JIA patients. Studies suggest that an acyl/free carnitine ratio above 0.4 is a supportive finding for carnitine deficiency [28]. In our study, neither JIA patients nor control subjects have high acyl/free carnitine ratio. It is interesting that JIA patients had lower total acylcarnitine levels, despite the high plasma levels of free carnitine and long-chain acyl-carnitines. The above-mentioned changes in carnitine homeostasis were suggested to be relevant with possible impairment of peripheral carnitine usage and mitochondrial energy metabolism in some individuals with JIA.

Pharmacological treatment of JIA consists of several options. Nonsteroidal antiinflammatory drugs (NSAIDs), steroids, and intra-articular injections with triamcinolone hexacetonide can be mentioned as first-line therapeutic agents. Methotrexate is a second-line agent for persistent arthritis. Several biologic agents are used in drug-resistant JIA, including etanercept (TNF-soluble receptor), Adalimumab (humanised anti-TNF antibody), Abatacept (T-cell co-stimulation inhibitor), Anakinra (IL-1 receptor antagonist), Canakinumab (Humanised anti-IL-1 antibody) [25]. The main goal of treatment is to control the inflammatory process. In our study, most of the patients were receiving antiinflammatory treatment; only four patients were refractory to treatment and 19 patients had not been receiving any medical treatment. Good control of autoinflammation by pharmacotherapy might have led to a lower need for carnitine in JIA patients. On the other hand, patients who received antiinflammatory treatment had higher levels of free carnitine than those who did not. These results may also indicate an impairment of long-chain fatty acid oxidation and free carnitine consumption by the antiinflammatory treatment. Also, another reason for the high carnitine levels in patients receiving antiinflammatory therapy may also be the correction of increased carnitine consumption triggered by inflammation with treatment.

The study group in our study was relatively small but included a variety of combinations in which therapeutic agents including etanercept, methotrexate, or steroids (46, 52, and 54 patients respectively) did not cause carnitine deficiency. Among 114 patients, 33 patients had complaints of fatigue, which may be associated with impaired energy metabolism. Elevated free carnitine levels in JIA patients have also been associated with insidious skeletal damage.

This study has some limitations. Most of the JIA patients received antiinflammatory treatment during the study. Antiinflammatory treatment may affect carnitine levels. In addition, the nutritional status of the JIA patients was not determined, especially regarding appropriate meat consumption.

In conclusion, we did not find secondary carnitine deficiency in JIA patients in this study, therefore carnitine supplementation is not recommended in all JIA patients. In contrast, alterations in free and acyl-carnitine levels were found in JIA patients. Therefore, impaired mitochondrial fatty acid oxidation could be a secondary problem in JIA patients. Further studies on screening carnitine status and fatty acid oxidation in JIA patients are warranted, especially screening carnitine status before and after treatment with antiinflammatory medications will prevent the actual carnitine status of JIA patients and the underlying factors that influence energy metabolism.

## Figures and Tables

**Table 1:** Demographic and clinic characteristics of JIA patients and control subjects.

Variables	Patients	Controls
**Sex (male/female)**	50/64	23/27
**Age (months)**	129.8 ± 53.6	129.67 ± 51.25
**Consanguinity**	59/114 (51.7%)	
**JIA Subtypes**		
**Oligoarticular JIA**	55/114 (48.2%)	
**Polyarticular JIA**	43/114 (37.7%)	
**Enthesitis related JIA**	10/114 (8.7%)	
**Systemic JIA**	6/114 (5.2%)	
**Responsive to medication**	110/114 (96.4%)	
**Fatigue**	33/114 (28.9%)	

**Table 2:** Free carnitine and acyl-carnitine levels of JIA patients and control subjects.

	JIA patients (μmol/L)	Control subjects (μmol/L)	p
**Free carnitine**	46.7671 ± 11.8631	33.0550 ± 7.8001	0.000
**Short-chain acyl-carnitines**			
**C2**	5.7534 ± 2.6138	15.2324 ± 4.5400	**0.000**
**C3**	1.0382 ± 0.4174	1.8066 ± 0.8142	**0.000**
**C4**	0.1556 ± 0.0633	0.2156 ± 0.0774	**0.000**
**C5**	0.1094 ± 0.0357	0.1056 ± 0.0305	0.511
**C5:1**	0.0155 ± 0.0073	0.0188 ± 0.0084	0.380
**Medium-chain acyl-carnitines**			
**C6**	0.0451 ± 0.0133	0.0638 ± 0.0207	**0.000**
**C8**	0.0625 ± 0.0280	0.0886 ± 0.0437	**0.000**
**C10**	0.1010 ± 0.0505	0.1364 ± 0.0715	**0.000**
**C10:1**	0.0890 ± 0.0383	0.1290 ± 0.0591	**0.000**
**C10:2**	0.0175 ± 0.0079	0.0216 ± 0.0084	**0.004**
**Long-chain acyl-carnitines**			
**C14**	0.1148 ± 0.0390	0.0862 ± 0.0250	**0.000**
**C14:1**	0.0587 ± 0.0231	0.0640 ± 0.0279	0.214
**C14:2**	0.0273 ± 0.0124	0.0228 ± 0.0127	**0.040**
**C16**	0.9424 ± 0.3137	0.7810 ± 0.2147	**0.001**
**C16:1**	0.0427 ± 0.0189	0.0444 ± 0.0166	0.578
**C18**	0.5726 ± 0.1955	0.4918 ± 0.1584	**0.011**
**C18:1**	0.8191 ± 0.2685	0.7662 ± 0.2188	0.225
**C18:2**	0.3501 ± 0.1383	0.3294 ± 0.0921	0.337
**Acyl-carnitine esters derived from dicarboxylic acids**			
**C3DC**	0.0300 ± 0.0128	0.0414 ± 0.0226	**0.000**
**C4DC**	0.2794 ± 0.0956	0.4268 ± 0.1497	**0.000**
**C5DC**	0.0266 ± 0.0101	0.0424 ± 0.0191	**0.000**
**Acyl-carnitine esters derived from hydroxylated acids**			
**C4-OH**	0.0399 ± 0.0156	0.0760 ± 0.0472	**0.000**
**C5-OH**	0.1986 ± 0.0957	0.2180 ± 0.0772	0.211
**C16-OH**	0.0224 ± 0.0098	0.0170 ± 0.0067	**0.001**
**C18:1-OH**	0.0117 ± 0.0048	0.0140 ± 0.0057	**0.011**
**C18:2-OH**	0.0126 ± 0.0079	0.0110 ± 0.0070	0.235
**Total acyl-carnitine**	5.1828 ± 1.2495	6.0280 ± 1.3345	**0.000**

Values are mean ± SD.

## References

[1] Martini A Lovell DJ Juvenile idiopathic arthritis: state of the art and future perspectives Annals of the Rheumatic Diseases 2010 69 7 1260 1263 10.1136/ard.2010.133033 20525835

[2] Moncrieffe H Prahalad S Thompson SD Genetics of juvenile idiopathic arthritis: new tools bring new approaches Current Opinion in Rheumatology 2014 26 5 579 584 10.1097/BOR.0000000000000094 25010442 PMC4445412

[3] Petty RE Southwood TR Manners P Baum J Glass DN International League of Associations for Rheumatology International League of Associations for Rheumatology classification of juvenile idiopathic arthritis: second revision, Edmonton, 2001 The Journal of Rheumatology 2004 31 2 390 392 14760812

[4] Kessler EA Becker ML Therapeutic advancements in juvenile idiopathic arthritis Best Practice & Research: Clinical Rheumatology 2014 28 2 293 313 10.1016/j.berh.2014.03.005 24974064

[5] Backström M Tynjälä P Ylijoki H Aalto K Kärki J Finding specific 10-joint Juvenile Arthritis Disease Activity Score (JADAS10) and clinical JADAS10 cut-off values for disease activity levels in non-systemic juvenile idiopathic arthritis: a Finnish multicentre study Rheumatology (Oxford) 2016 55 4 615 623 10.1093/rheumatology/kev353 26447164

[6] Walter JH L-Carnitine Archives of Disease in Childhood 1996 74 6 475 478 10.1136/adc.74.6.475 8758120 PMC1511567

[7] Stanley CA Carnitine deficiency disorders in children Annals of the New York Academy of Sciences 2004 1033 42 51 10.1196/annals.1320.004 15591002

[8] Mescka C Moraes T Rosa A Mazzola P Piccoli B In vivo neuroprotective effect of L-carnitine against oxidative stress in maple syrup urine disease Metabolic Brain Disease 2011 26 1 21 28 10.1007/s11011-011-9238-x 21380499

[9] Li JL Wang QY Luan HY Kang ZC Wang CB Effects of L-carnitine against oxidative stress in human hepatocytes: involvement of peroxisome proliferator-activated receptor alpha Journal of Biomedical Science 2012 21 19 1 32 10.1186/1423-0127-19-32 22435679 PMC3338374

[10] Garcia CL Filippi S Mosesso P Calvani M Nicolai R Mosconi L Palitti F The protective effect of L-carnitine in peripheral blood human lymphocytes exposed to oxidative agents Mutagenesis 2006 21 1 21 27 10.1093/mutage/gei065 16306135

[11] Khalatbari-Soltani S Tabibi H Inflammation and L-carnitine therapy in hemodialysis patients: a review Clinical and Experimental Nephrology 2015 19 3 331 335 10.1007/s10157-014-1061-3 25446285

[12] Tedgui A The role of inflammation in atherothrombosis: implications for clinical practice Vascular Medicine 2005 10 1 45 53 10.1191/1358863x05vn589ra 15921000

[13] Szmitko PE Wang CH Weisel RD de Almeida JR Anderson TJ Verma S New markers of inflammation and endothelial cell activation: Part I Circulation 2003 21 108 16 1917 1923 10.1161/01.CIR.0000089190.95415.9F 14568885

[14] Engel AG Angelini C Carnitine deficiency of human skeletal muscle with associated lipid storage myopathy: a new syndrome Science 1973 2 179 4076 899 902 10.1126/science.179.4076.899 4687787

[15] Roe CR Millington DS Kahler SG Kodo N Norwood DL Carnitine homeostasis in the organic acidurias Progress in clinical and biological research 1990 321 383 402 2183238

[16] Rinaldo P Matern D Bennett MJ Fatty acid oxidation disorders Annual Review of Physiology 2002 64 477 502 10.1146/annurev.physiol.64.082201.154705 11826276

[17] Lheureux PE Penaloza A Zahir S Gris M Science review: carnitine in the treatment of valproic acid-induced toxicity - what is the evidence? Critical Care 2005 5 9 5 431 440 10.1186/cc3742 16277730 PMC1297603

[18] Holme E Greter J Jacobson CE Lindstedt S Nordin I Carnitine deficiency induced by pivampicillin and pivmecillinam therapy Lancet 1989 26 2 8661 469 473 10.1016/s0140-6736(89)92086-2 2570185

[19] Veselá E Racek J Trefil L Jankovy’ch V Pojer M Effect of L-carnitine supplementation in hemodialysis patients Nephron 2001 88 3 218 223 10.1159/000045993 11423752

[20] Sakurauchi Y Matsumoto Y Shinzato T Takai I Nakamura Y Effects of L-carnitine supplementation on muscular symptoms in hemodialyzed patients American Journal of Kidney Diseases 1998 32 2 258 264 10.1053/ajkd.1998.v32.pm9708610 9708610

[21] Uysal N Yalaz G Acikgoz O Gonenc S Kayatekin BM Effect of L-carnitine on diabetogenic action of streptozotocin in rats Neuro Endocrinology Letters 2005 26 4 419 22 16135998

[22] Rezaee H Khalili H Salamzadeh J Jafari S Abdollahi A Potential benefits of carnitine in HIV-positive patients Future Virology 2012 7 1 73 83 10.2217/fvl.11.133

[23] Bonafé L Berger MM Que YA Mechanick JI Carnitine deficiency in chronic critical illness Current Opinion in Clinical Nutrition & Metabolic Care 2014 17 2 200 209 10.1097/MCO.0000000000000037 24500444

[24] Hatamkhani S Karimzadeh I Elyasi S Farsaie S Khalili H Carnitine and sepsis: a review of an old clinical dilemma Journal of Pharmaceutical Sciences 2013 16 3 414 423 10.18433/j3js4c 24021290

[25] Stoll ML Cron RQ Treatment of juvenile idiopathic arthritis: a revolution in care Pediatric Rheumatology Online Journal 2014 23 12 13 10.1186/1546-0096-12-13 PMC4003520 24782683

[26] Schulze A Lindner M Kohlmüller D Olgemöller K Mayatepek E Hoffmann GF Expanded newborn screening for inborn errors of metabolism by electrospray ionization-tandem mass spectrometry: results, outcome, and implications Pediatrics 2003 111 6 Pt 1 1399 1406 10.1542/peds.111.6.1399 12777559

[27] Millington DS Norwood DL Kodo N Roe CR Inoue F Application of fast atom bombardment with tandem mass spectrometry and liquid chromatography/mass spectrometry to the analysis of acylcarnitines in human urine, blood, and tissue Analytical Biochemistry 1989 1 180 2 331 339 10.1016/0003-2697(89)90441-7 2817363

[28] Reuter SE Evans AM Carnitine and acylcarnitines: pharmacokinetic, pharmacological and clinical aspects Clinical Pharmacokinetics 2012 1 51 9 553 572 10.1007/BF03261931 22804748

